# Fecal and Serum Calprotectin Concentrations in Cats With Chronic Enteropathies Before and During Treatment

**DOI:** 10.1111/jvim.70067

**Published:** 2025-03-20

**Authors:** Dimitra A. Karra, Jonathan A. Lidbury, Jan S. Suchodolski, Matina Pitropaki, Shelley Newman, Jeorg M. Steiner, Panagiotis G. Xenoulis

**Affiliations:** ^1^ Clinic of Medicine, Faculty of Veterinary Science University of Thessaly Karditsa Greece; ^2^ Gastrointestinal Laboratory, Department of Small Animal Clinical Sciences Texas A&M University College Station Texas USA; ^3^ Newman Specialty VetPath Hicksville New York USA

**Keywords:** biomarker, chronic inflammatory enteropathy, gastrointestinal, lymphoma

## Abstract

**Background:**

Chronic enteropathies (CE) are common in cats. Reliable biomarkers that can distinguish different causes and predict or monitor responses to treatment are currently lacking.

**Hypothesis/Objectives:**

Evaluate calprotectin concentrations in serum and feces as potential biomarkers in cats with CE.

**Animals:**

Forty‐three cats with either chronic inflammatory enteropathy (CIE; *n* = 25) or small cell gastrointestinal lymphoma (SCGL; *n* = 18) and 36 healthy cats were prospectively enrolled.

**Methods:**

Fecal and serum calprotectin concentrations were determined before and during treatment. Cats with CIE were treated with diet, prednisolone, or diet and prednisolone, and cats with SCGL were treated with prednisolone plus chlorambucil with or without diet.

**Results:**

Compared to controls, fecal calprotectin concentration was significantly higher in cats with CE (median, ≤ 161 ng/g; range, ≤ 161–2827 vs. median, ≤ 161 ng/g; range, ≤ 161–790; *p* = 0.01). No significant differences were found in fecal (median ≤ 161 ng/g; range, ≤ 161–1920 vs. median, 189 ng/g; range, ≤ 161–2827; *p* = 0.3) or serum calprotectin (median, ≤ 1291 mg/L; range, ≤ 1291–15 358 vs. median, ≤ 1291 mg/L; range, ≤ 1291–6422; *p* = 0.99) between cats with CIE and cats with SCGL. Fecal calprotectin was significantly decreased after treatment in cats with CE (median, ≤ 161 ng/g; range, ≤ 161–1897 vs. median, ≤ 161 ng/g; range, ≤ 161–656; *p* = 0.02).

**Conclusions:**

Fecal calprotectin concentration might be a good biomarker for diagnosis and treatment monitoring for a subset of cats with CE. Serum calprotectin concentrations do not seem to be useful for diagnosis or treatment monitoring in cats with CE. Neither fecal nor serum calprotectin concentrations could differentiate cats with CIE from cats with SCGL.

**Abbreviations**
CE: chronic enteropathies
CIE: chronic inflammatory enteropathy
CKD: chronic kidney disease
DLH: domestic longhair
DSH: domestic shorthair
FCEAI: feline chronic enteropathy activity index
FRE: food responsive enteropathy
GI: gastrointestinal
IRE: immunoressant responsive enteropathy
IRIS: International Renal Interest Society
SCGL: small cell gastrointestinal lymphoma
WSAVA: World Small Animal Veterinary Association

## 1 Introduction

Chronic enteropathy (CE) in cats is a collective term used to describe a diverse group of gastrointestinal (GI) diseases in cats that result in chronic (longer than 3 weeks' duration) clinical signs of intestinal dysfunction, such as decreased appetite or anorexia, diarrhea, vomiting, weight loss, or some combination of these signs. Chronic enteropathies have been categorized into chronic inflammatory enteropathy (CIE) and small cell GI lymphoma (SCGL) [1, 2, 3, 4, 5, 6]. Further subclassification of CIE is based on response to treatment and is divided into immunosuppressant‐responsive enteropathy (IRE) and food‐responsive enteropathy (FRE) [1]. Small cell GI lymphoma is the most common GI neoplasm in cats, and its prevalence has increased during the past two decades [7, 8]. Progression of CIE to SCGL over months to years has long been suspected, but this progression has not been definitively proven as of yet [9, 10]. Currently, establishment of a definitive diagnosis of CIE or SCGL is based on histopathology with immunohistochemistry and sometimes clonality testing [5, 9, 11, 12, 13].

The ability to predict response to treatment and monitor disease activity and treatment efficacy with non‐invasive tools is highly desirable. In 2001, the Biomarker Definitions Working Group defined a biomarker as a characteristic that is objectively measured and evaluated as an indicator of normal biological processes, pathologic processes, or pharmacologic response to a therapeutic intervention [14]. In inflammatory conditions, proteins, such as calprotectin, are commonly released by inflammatory cells or in response to tissue dysfunction, and some of them may serve as biomarkers [15, 16].

In humans with Crohn's disease, evaluation and monitoring of response to treatment by use of a tight control algorithm that includes both clinical signs and inflammatory biomarkers increases the possibility of disease remission compared with only using clinical signs for treatment monitoring [17]. In cats with CE, noninvasive biomarkers for treatment monitoring have not been systematically evaluated [16].

Calprotectin is a protein that belongs to the damage‐associated molecular pattern (DAMP) molecules of the innate immune response. Although the development and validation of assays for the measurement of calprotectin concentrations in cat feces and serum have been reported, their utility as biomarkers in treatment monitoring in cats with CE has only been poorly evaluated to date [18]. In a recent study, fecal calprotectin was evaluated in cats with CIE and SCGL [19], and in a more recent study, fecal calprotectin was evaluated before and after treatment in 17 cats with CE [20]. The results support the utility of fecal calprotectin as a surrogate biomarker to assess disease severity in cats with CE [20]. No studies have evaluated serum calprotectin concentration in cats with CE before and after treatment.

Our hypothesis was that fecal and serum calprotectin concentrations could be used as biomarkers for the diagnosis, prediction of response to treatment, and monitoring of treatment in cats with CE. Therefore, our aims were: (a) to measure serum and fecal calprotectin concentrations in cats with CE and compare them with those of healthy control cats; (b) to compare fecal and serum calprotectin concentrations between cats with CIE and those with SCGL; and (c) to evaluate changes in fecal and serum calprotectin concentrations before and during treatment in cats with CE.

## 2 Materials and Methods

### 2.1 Ethics Statement

The study protocol was reviewed and approved by the Animal Ethics Committee of the University of Thessaly, Greece (AUP number:115/6.10.2020). The owner of each cat enrolled in the study signed an informed owner consent form.

### 2.2 Cats

Cats presented from September 2020 to October 2022 to the Clinic of Medicine of the University of Thessaly and a referral clinic in Athens, Greece, for persistent or intermittent GI signs of at least 3 weeks' duration were considered for inclusion in the study. Written informed client consent was collected before patient enrollment. Cats were included in the study if (1) they had at least one clinical sign compatible with CE (diarrhea or loose feces of > 3 weeks' duration; vomiting ≥ 2 times per month for ≥ 2 months, weight loss, hyporexia, anorexia or some combination of these signs); (2) had undergone gastroduodenoscopy and ileocolonoscopy with biopsy and histopathologically confirmed CIE or SCGL (see below); (3) had no other diseases that could explain the cats' clinical signs; and (4) had no history of administration of antibiotics, corticosteroids, or other immunosuppressive agents, such as chlorambucil or cyclosporine, for at least 3 weeks before presentation, to exclude any potential impact on histopathology results. Cats with a clinical presentation compatible with CE that had concurrent diseases that were well controlled with appropriate treatment were also eligible for enrollment. Cats with concurrent pancreatitis, inflammatory liver disease, or both also were enrolled in the study. Strictly indoor, dewormed cats at least 1 year of age without a prior or current history of chronic GI signs or antibiotic or corticosteroid administration for at least 1 year, with no abnormal findings on physical examination and with negative PCR for *Tritrichomonas blagburni* were recruited as healthy controls.

### 2.3 Study Design and Fecal and Serum Sample Collection

All cats with CE underwent the same diagnostic investigations, including a complete history, physical examination, and a minimum of CBC, serum biochemistry profile with electrolytes and serum cobalamin, folate, pancreatic lipase immunoreactivity (measured as Spec fPL, IDEXX) [21] and trypsin‐like immunoreactivity (TLI) [22] concentrations, urinalysis, fecal parasitology using saturated zinc sulfate flotation, PCR for *Tritrichomonas blagburni*, abdominal ultrasound examination, upper and lower GI endoscopy, and biopsy collection for histopathology and immunochemistry examination. The feline chronic enteropathy activity index (FCEAI) [3] also was calculated for all cats with CE. Re‐examinations with measurement of CBC, serum biochemistry with electrolytes, and serum cobalamin, folate, Spec fPL, and fTLI concentrations were performed on day 90 after endoscopy.

Endoscopy was performed by a veterinarian with experience in endoscopy using a flexible video endoscope (Pentax EG2490Κ, PENTAX Medical EMEA) with an outer diameter of 8 mm and a 2.4 mm biopsy channel. Biopsy samples were obtained using a 2.2 mm forceps. Before endoscopy, food was withheld for 12 h, and 4–5 warm water enemas were performed to cleanse the colon while the cat was under general anesthesia. At each endoscopy, at least 5 high‐quality biopsy samples were collected from the stomach, 10 from the duodenum, 4 from the ileum, and 5 from the colon. Feces collected during the enema and blood were collected at baseline (T0; before endoscopy). Naturally passed feces and blood also were collected 90 days (T1) after endoscopy. Single naturally passed fecal and serum samples also were collected from cats in the control group. All fecal and serum samples were placed in Eppendorf tubes and stored at −80°C until further analysis.

Complete blood count and serum biochemistry profile with electrolytes was performed using in‐house analyzers (Vetscan HM5 Hematology Analyzer and Vetscan VS2 Chemistry Analyzer). Serum concentrations of cobalamin, folate, Spec fPL, and fTLI were measured at the Gastrointestinal Laboratory at the College of Veterinary Medicine & Biomedical Sciences (VMBS) at Texas A&M University.

### 2.4 Histopathology and Immunohistochemistry

Formalin‐fixed, paraffin‐embedded endoscopic biopsy samples were stained with hematoxylin and eosin (H&E) for histopathologic examination. Endoscopic biopsy samples were examined by a board‐certified pathologist with expertise in small animal GI histopathology. Findings were reported descriptively and numerically scored according to the World Small Animal Veterinary Association (WSAVA) histopathologic scoring system [23]. Both inflammatory (presence of lymphocytes, plasma cells, eosinophils, neutrophils, and macrophages in the lamina propria) as well as morphological (e.g., surface epithelial injury, crypt lesions such as dilatation, distortion, or hyperplasia, atrophy, and fibrosis) features were assessed histologically and assigned a score (normal = 0, mild = 1, moderate = 2, and marked = 3). Immunohistochemistry was conducted using a stepwise approach. Staining for T‐, B‐, and natural killer (NK) cell markers (CD3, CD79a, and granzyme B, respectively) was performed if deemed necessary based on the pathologist's discretion depending on results of H&E staining (i.e., number, size, and distribution of mucosal lymphocytes). Based on pathologist's discretion, clonality testing (PARR) also was performed.

### 2.5 Assays for Fecal and Serum Calprotectin Concentrations

Serum and fecal calprotectin concentrations were measured using a species‐specific ELISA that has been analytically validated for use in cats [18]. The detection limit for the serum assay was 1291 mg/L and for the fecal assay 161.3 ng/g feces. Spot fecal samples were collected from all cats at the time of the first visit and at 90 days after endoscopy (T1) during the second re‐examination. Samples were stored frozen (–80°C) until analysis. Fecal samples then were thawed and extracted, and biomarker concentrations were measured in two batches, each containing a mixture of samples from CE cats and control samples. Samples were run in duplicate alongside internally produced quality control samples.

### 2.6 Clinical Activity Index

The clinical activity before and after treatment was evaluated using the FCEAI [3]. The FCEAI is based on five clinical criteria (each scored on a scale of 0–3: attitude/activity, appetite, vomiting, fecal consistency, and weight loss) and evaluation of three biochemistry variables (each scored with 0 when absent or 1 if present: increased total protein concentration, increased ALT activity, increased ALP activity, and decreased phosphorus concentration). The criterion endoscopic lesions was omitted from the calculation of the FCEAI because cats did not undergo repeated endoscopy [20, 24].

### 2.7 Treatments in Cats With CE

All cats received the same preventative antiparasitic treatment (Broadline, Boehringer Ingelheim) for the duration of the study. After endoscopy and biopsy sample collection, owners were advised to feed their cats the same hydrolyzed protein diet (Anallergenic, Royal Canin). All cats had been on at least one, but usually more, additional dietary trials by their referring veterinarian before endoscopy. In addition, all cats received hydroxycobalamin injections (250 μg/cat SC every 2 weeks) for the duration of the study. Cats with a diagnosis of CIE that did not respond to the study diet or did not consume the study diet were treated with prednisolone (these cats were diagnosed as IRE; starting dosage 2 mg/kg, PO q24h, with gradual tapering), whereas cats with a diagnosis of SCGL were treated with prednisolone (as for cats diagnosed with IRE) plus chlorambucil (2 mg/cat PO every 2nd or 3rd day). Immunomodulatory treatment was started 14 to 30 days after endoscopy, depending on the severity of the disease and the time needed to obtain histopathology results. Cats that fully responded to the hydrolyzed protein diet received no pharmacological treatment. If it was deemed necessary by the attending veterinarian, cats could receive symptomatic treatment during the first days of the treatment (e.g., maropitant, proton‐pump inhibitors, appetite stimulants).

### 2.8 Statistical Analysis

For statistical analysis, fecal calprotectin concentrations and serum calprotectin concentrations that were below the limit of detection of the assay were reported as 161.3 ng/g and 1290 mg/L, respectively. Data were tested for normality using the Shapiro–Wilk test. Normally distributed data were compared using *t* tests, whereas non‐normally distributed data were evaluated using the Mann Whitney test or Kruskal‐Wallis test. Fecal and serum calprotectin concentrations at T0 between the healthy control and CE groups were compared using the Mann Whitney test. Fecal and serum calprotectin concentrations between healthy control, CIE, and SCGL groups were compared using the Kruskal‐Wallis test. Fecal and serum calprotectin concentrations before and during treatment were compared using the Wilcoxon test. Spearman correlation (*r*) was used to assess the relationship between fecal and serum calprotectin concentrations and the FCEAI. Significance was set at *p* < 0.05. All data were analyzed using commercially available statistical software (GraphPad Prism 8).

## 3 Results

A total of 43 cats with CE were included in the study. Of these, feces were available from 41 cats and serum from 40 cats at baseline. Based on clinical response, histopathology, and immunochemistry results, 25 cats were diagnosed with CIE (58%), (19 IRE [76%] and 6 FRE [24%]), and 18 with SCGL (42%; Table 1).

**TABLE 1:** Characteristics of cats with CE and subgroups CIE and SCGL at baseline.

Variable	CE (*n* = 43)	CIE (*n* = 25)	SCGL (*n* = 18)
Male	25 (58%)	16 (64%)	9 (50%)
Female	18 (42%)	9 (36%)	9 (50%)
Age (years)	11 (3–16)	9 (3–15)	12.5 (3–16)
Body weight (kg)	4.6(1.5)	4.6 (1.2)	4.6 (1.8)
FCEAI	8 (4–15)	7 (4–13)	9 (4–15)
Decreased activity	22 (51%)	12 (48%)	10 (55%)
Decreased appetite	25 (58%)	14 (56%)	11 (61%)
Vomiting	39 (90%)	22 (88%)	17 (94%)
Diarrhea	31 (72%)	16 (64%)	15 (83%)
Weight loss	36 (84%)	21 (56%)	10 (55%)
Concurrent diseases			
Pancreatitis	3 (7%)	2 (8%)	1 (6%)
Hyperthyroidism	4 (9%)	1 (4%)	3 (17%)
CKD II	2 (5%)	2 (8%)	
Diabetes mellitus	2 (5%)	2 (8%)	
Stomatitis	3 (7%)	2 (8%)	1 (6%)
Idiopathic hypertension	1 (2%)		1 (6%)

Abbreviations: CE, chronic enteropathy; CIE, chronic inflammatory enteropathy; CKD, chronic kidney disease; FCEAI, feline chronic enteropathy activity index; SCGL, small cell gastrointestinal lymphoma.

### 3.1 Concurrent Diseases

At initial presentation, of the 43 cats with CE included in the study, three cats (2 with IRE and 1 with SCGL) had concurrent pancreatitis (based on an increased serum Spec fPL concentration ≥ 8.8 μg/L), four (1 with IRE and 3 with SCGL) had hyperthyroidism that was under control using medical treatment, three (all with IRE) had stable International Renal Interest Society (IRIS) stage II chronic kidney disease (CKD), three (2 with IRE, 1 with SCGL) had stomatitis, two cats had diabetes mellitus that was under control using medical treatment, one (with SCGL) had idiopathic hypertension and was being managed with amlodipine, and one cat with IRE had asthma and was being treated with inhaled fluticasone (Table 1).

### 3.2 Clinical Signs

The most common presenting clinical sign in cats with CE was vomiting (39/43; 91%), followed by weight loss (36/43; 84%), diarrhea (31/43; 72%), and hyporexia or anorexia (25/43; 58%;Table 1). In cats with CIE, the most common clinical signs were vomiting (21/24, 87%), weight loss (20/24, 83.3%), diarrhea (15/24, 62.5%), and hyporexia or anorexia (13/24, 54%). In cats with SCGL, the most common clinical signs were vomiting (18/19, 94%), weight loss (16/19, 84%), diarrhea (16/19, 84.2%), and hyporexia or anorexia (12/19, 63%;Table 1).

### 3.3 Treatment and Follow‐Up

Of the 43 cats with CE included in the study, follow‐up information was available for 28 cats at T1 (Day 90). Of these, 11 had IRE, 6 had FRE, and 11 had SCGL. All cats were switched to the same hydrolyzed protein diet after endoscopy (Anallergenic, Royal Canin). However, only 20 of 28 cats consumed the diet exclusively, whereas one cat consumed Anallergenic together with hypoallergenic (Royal Canin) canned food. In two cats, owners reported that clinical signs worsened after switching to Anallergenic, with one cat developing watery diarrhea and the other experiencing a worsening of vomiting and diarrhea that led the owners to switch to their previous diets (Purina One Sterilized dry food and Applaws Grain‐Free wet food, respectively). In another cat, the diet was not changed because of the risk of dysregulation of the cat's diabetes mellitus that was well controlled with diet and insulin. One cat at re‐examination started a diet for cats with renal disease because of the concurrent development of CKD. The remaining 4 cats continued to consume their previous diet because of their refusal to consume the study diet.

Cats with IRE had been treated with prednisolone for 30–45 days at T1. Cats with SCGL had been treated with prednisolone and chlorambucil for 30–45 days at T1. In cats with CE, mean FCEAI was 8.5 (SD, 2.7; *n* = 43) at T0 and 3.5 (SD, 2; *n* = 28) at T1. For cats with CE for which follow‐ up information was available, mean FCEAI was significantly decreased at T1 (mean, 3.5; SD, 2.0; *n* = 28) compared to T0 (mean, 8.7; SD, 2.8; *n* = 28 *p* < 0.001). During the study period, one cat with CIE‐IRE had been treated by the owner with ondansetron for 10 days before the initiation of prednisolone. Another cat additionally received mirtazapine because of prolonged hyporexia and weight loss.

### 3.4 Fecal Calprotectin

#### 3.4.1 Signalment of CE Cats

Feces from 41 cats with CE were collected for measurement of calprotectin concentration (fCE‐group). Twenty‐nine cats from the fCE‐group were domestic shorthair (DSH), 5 were domestic longhair (DLH), 2 were Birman, 2 were Persian, 1 was Norwegian Forest, 1 was Ragdoll, and 1 was Bengal. Cats of the fCE‐group had a mean body weight of 4.6 kg (SD, 1.4) and a median age of 11 years (range, 3–16). Twenty‐four cats of the fCE‐group were male (23 neutered) and 17 were female (all sprayed).

#### 3.4.2 Signalment of Healthy Controls

Fecal samples from 23 healthy cats were collected to be used as controls for the determination of fecal calprotectin concentration (fHC‐group). All cats of the fHC‐group were DSH, had a mean body weight of 4.5 kg (SD 1.5) and a mean age of 2.9 years (SD 1.1). Ten were male (all neutered) and 13 were female (all spayed). Mean body weight was not significantly different between cats in the fCE‐group and cats in the fHC‐group. However, the ages between cats in the fCE‐group and cats in the fHC‐group were significantly different (11 vs. 3 years; *p* < 0.001).

### 3.5 Fecal Calprotectin Concentrations in Cats With CE and Healthy Cats at Baseline

**FIGURE 1:**
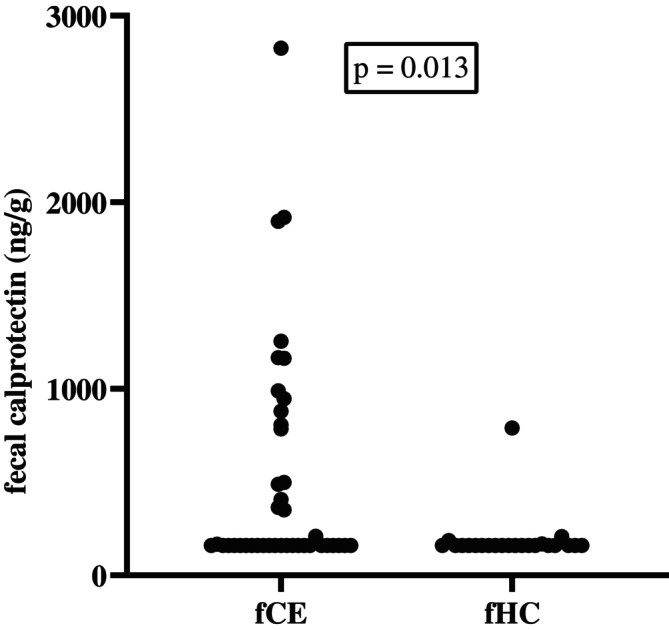
Fecal calprotectin concentrations in cats with CE and healthy control cats (fHC) at baseline. A significant difference in fecal calprotectin concentrations was found between the two groups at baseline (*p* = 0.01). Several cats had the same fecal calprotectin concentrations (because lower detection limit of the assay) and therefore they are not clearly separated in the graph.

Twenty‐three of 41 cats (56%) in the fCE‐group had fecal calprotectin concentrations below the detection limit of the assay (161 ng/g). The medians (ranges) of fecal calprotectin concentrations in the fCE‐group at baseline and the fHC‐group were ≤ 161 ng/g (≤ 161–2827) and ≤ 161 ng/g (≤ 161–790), respectively. A significant difference in fecal calprotectin concentrations in the fCE‐group was observed compared with the fHC‐group (*p* = 0.01; Figure 1).

### 3.6 Fecal Calprotectin Concentrations in Cats With CIE and Cats With SCGL at Baseline

**FIGURE 2:**
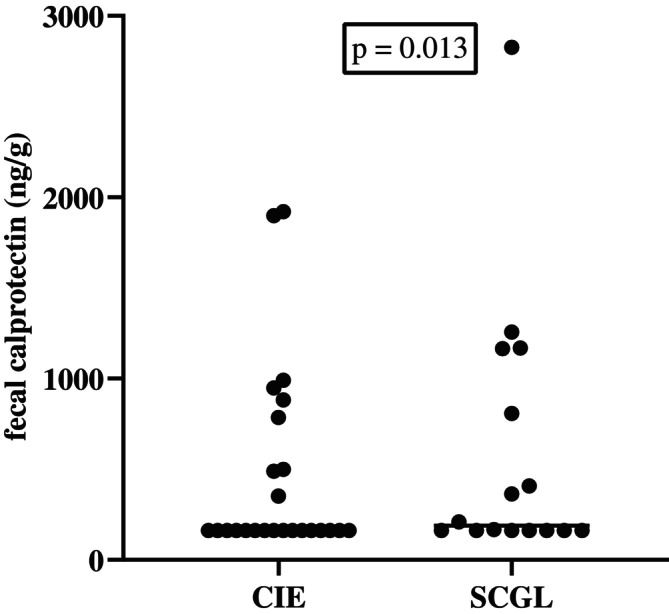
Fecal calprotectin concentrations in healthy control cats (fHC), in cats with CIE, and in cats with SCGL at baseline. No significant difference in fecal calprotectin concentrations was found between cats with CIE and cats with SCGL at baseline (*p* = 0.67). No significant difference in fecal calprotectin concentrations was found between cats with CIE and fHC cats (*p* = 0.26). A statistically significant difference was observed between fHC cats and cats with SCGL (*p* = 0.02). Several cats had the same fecal calprotectin concentrations (because of lower detection limit of the assay) and therefore, they are not clearly separated in the graph.

Twenty‐five cats of the fCE‐group were diagnosed with CIE (19, IRE; 6, FRE) and 16 with SCGL. Fecal calprotectin concentrations were not significantly different (*p* = 0.67) between cats with CIE (median, ≤ 161 ng/g; range, ≤ 161–1920) and cats with SCGL (median, 189 ng/g; range, ≤ 161–2827;Figure 2).

### 3.7 Fecal Calprotectin Concentrations in Cats With CE Before and During Treatment

**FIGURE 3:**
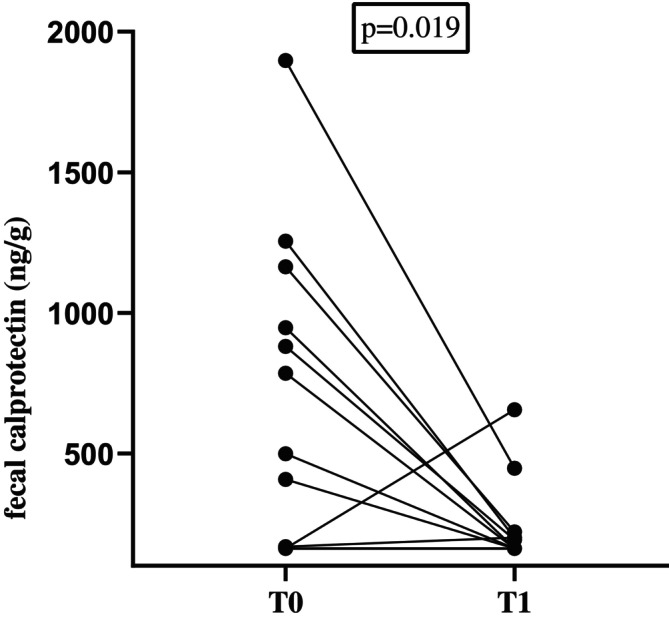
Fecal calprotectin concentrations in cats with CE before (T0) and after treatment (T1). A significant difference in fecal calprotectin concentrations was found between the two time points (*p* = 0.02). Several cats had the same fecal calprotectin concentrations (because of lower detection limit of the assay) and therefore they are not clearly separated in the graph. Many cats had fecal calprotectin concentrations below the limit of detection for both T0 and T1.

Fecal samples were available from 22 cats from the fCE‐group at T1 (15 cats with CIE and 7 with SCGL). A significant decrease from T0 to T1 was found in fecal calprotectin concentrations (median, ≤ 161 ng/g; range, ≤ 161–1897 vs. median, ≤ 161 ng/g; range, ≤ 161–656; *p* = 0.02;Figure 3).

### 3.8 Serum Calprotectin

#### 3.8.1 Signalment of CE Cats

Serum from 40 cats with CE was available for measurement of serum calprotectin concentrations (sCE‐group). Twenty‐nine of these cats were DSH, 5 were DLH, 2 were Birman, 1 was Norwegian Forest, 2 were Persian, and 1 was Ragdoll. Cats of the sCE‐group had a mean body weight of 4.5 kg (SD, 1.4) and a median age of 11 years (range, 3–16). Twenty‐three cats were male (22 neutered) and 17 were female (all sprayed).

#### 3.8.2 Signalment of Healthy Controls

Serum from 18 healthy control cats was collected for serum calprotectin measurement (sHC‐group). All cats in the sHC‐group were DSH, had a mean body weight of 5.3 kg (SD, 2.0) and a median age of 3 years (range, 1–11). Eleven were male (all neutered) and 7 were female (all spayed). Mean body weight was not significantly different between the sCE‐group and the sHC‐group. Ages between the sCE‐group and the sHC‐group were significantly different (11 vs. 3 years, *p* < 0.001).

### 3.9 Serum Calprotectin Concentrations in Cats With CE and Healthy Cats at Baseline

**FIGURE 4:**
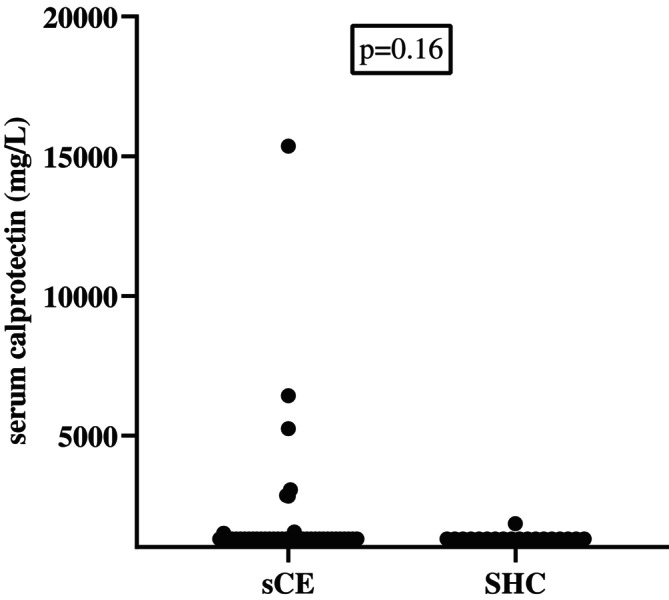
Serum calprotectin concentrations in cats with CE compared to healthy control (sHC) cats at baseline. No significant difference was found between the two groups. Several cats had the same serum calprotectin concentrations (because of lower detection limit of the assay) and therefore they are not clearly separated in the graph.

Thirty‐two cats of 40 (80%) in the sHC group had serum calprotectin concentrations below the detection limit of the assay (1291 mg/L). The medians (ranges) of serum calprotectin concentrations in the sCE group of cats at baseline and in the sHC group of cats were 1935 mg/L (≤ 1291–15 358) and 1321 mg/L (≤ 1291–1846), respectively. No significant difference in serum calprotectin concentrations was found between the sCE group and the sHC group (*p* = 0.16; Figure 4).

### 3.10 Serum Calprotectin Concentrations in Cats With CIE and Cats With SCGL at Baseline

**FIGURE 5:**
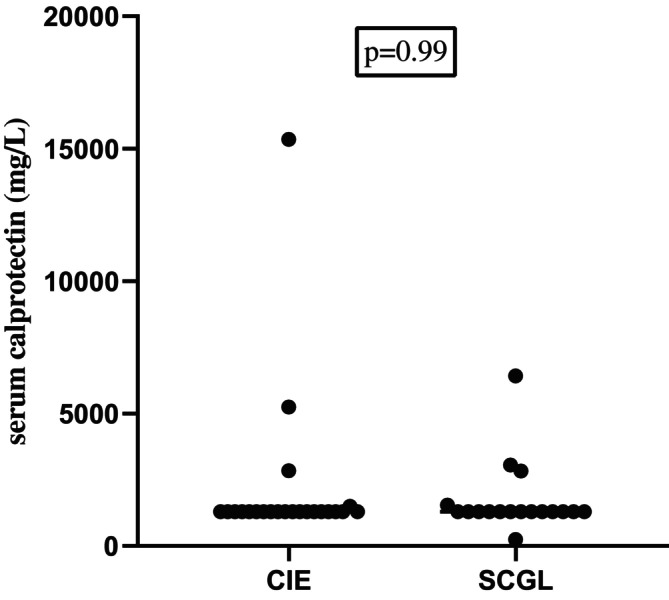
Serum calprotectin concentrations in healthy control (sHC) cats, in cats with CIE, and in cats with SCGL at baseline. No significant difference was found between cats with CIE and cats with SCGL (*p* = 0.99), neither between sHC cats and cats with CIE (*p* = 0.85) nor between sHC cats and cats with SCGL (*p* = 0.96). Several cats had the same serum calprotectin concentrations (because of lower detection limit of the assay) and therefore they are not clearly separated in the graph.

Twenty‐three cats of the sCE‐group were diagnosed with CIE (17, IRE; 6, FRE) and 18 with SCGL. Serum calprotectin concentrations were not significantly different (*p* = 0.99) between cats with CIE (median, ≤ 1291 mg/L; range, ≤ 1291–15 358) and those with SCGL (median, ≤ 1291 mg/L; range, ≤ 1291—6422; Figure 5).

### 3.11 Serum Calprotectin Concentrations in Cats With CE Before and During Treatment

**FIGURE 6:**
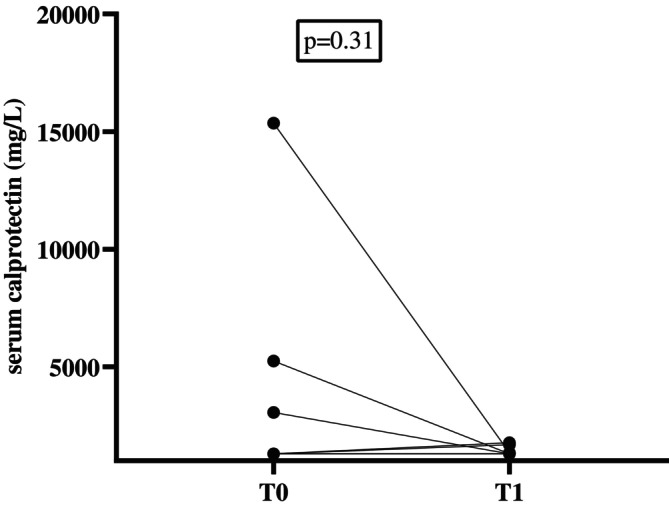
Serum calprotectin concentrations in cats before (T0) and after treatment (T1). no significant difference was found between the two timepoints (*p* = 0.31). Several cats had the same serum calprotectin concentrations and therefore they are not clearly separated in the graph. Many cats had serum calprotectin concentrations below the limit of detection for both T0 and T1.

Serum was available from 27 cats from the sCE group at T1 (16 cats with CIE and 11 with SCGL). No significant difference in serum calprotectin concentrations was found between sCE cats at T0 (median, ≤ 1291 mg/L; range, ≤ 1291–15 358) and T1 (median, ≤ 1291 mg/L; range, ≤ 1291—1762; *p* = 0.31; Figure 6).

### 3.12 Correlations Between Fecal and Serum Calprotectin Concentrations and FCEAI

The FCEAI before treatment was not correlated with fecal (Spearman *r* = 0.17; *p* = 0.31) or serum calprotectin concentrations (Spearman *r* = 0.16; *p* = 0.31). During treatment, FCEAI also did not correlate with either fecal (Spearman *r* = 0.04; *p* = 0.83) or serum (Spearman *r* = 0.31; *p* = 0.11) calprotectin concentrations.

### 3.13 Correlations Between Fecal and Serum Calprotectin, FCEAI, and Histopathological Scoring Grade

The correlation between FCEAI fecal and serum calprotectin concentration and summative histopathology score was not significant for either segment of the gastrointestinal tract. The correlation between the fecal calprotectin concentration and histopathology score was significant for stomach mucosal fibrosis (Spearman *r* = −0.41; *p* = 0.01). The correlation between the serum calprotectin concentration and histopathology score was significant for duodenum villous stunning (Spearman *r* = 0.33; *p* = 0.03), lacteal dilatation (Spearman *r* = 0.35; *p* = 0.03), lamina propria eosinophils (Spearman *r* = 0.33; *p* = 0.03), lamina propria neutrophils (Spearman *r* = 0.33; *p* = 0.03) and ileum epithelial injury (Spearman *r* = 0.32; *p* = 0.04). Despite statistical significance, correlations were weak to modest in all cases.

## 4 Discussion

In our prospective study, we investigated whether serum or fecal calprotectin concentrations could serve as biomarkers to differentiate the different forms of CE and monitor treatment in cats with CE.

In our study, fecal calprotectin concentrations were significantly increased at diagnosis in cats with CE, compared with healthy control cats. These findings are similar to those reported in a recent study in cats, in which cats with IRE, FRE, and SCGL had higher fecal calprotectin concentrations than healthy cats [19]. Our results are also similar to those described in two previous studies in dogs in which fecal calprotectin concentrations of dogs with inflammatory bowel disease (IBD) [25] or dogs with chronic diarrhea [26] were compared to fecal calprotectin concentrations in healthy dogs. In humans with IBD, fecal calprotectin is a promising biomarker and has been widely studied [27, 28]. Fecal calprotectin in humans can distinguish GI signs caused by organic disorders (such as IBD) from those resulting from functional disorders (e.g., irritable bowel syndrome) with high sensitivity and specificity [29]. Our results support the hypothesis that fecal calprotectin is increased in cats with CE compared with healthy controls, but both seemed to decrease with treatment. Whether or not either fecal or serum calprotectin concentration could serve as biomarkers for objectively monitoring disease activity in cats with either form of CE remains to be determined. Serum and fecal calprotectin concentrations failed to differentiate CIE enteropathies from SCGL.

In cats with CE, response to treatment generally is based on evaluation of clinical activity. Because repeated endoscopies and histopathological evaluations are unrealistic in clinical practice, biological markers that are able to indirectly evaluate GI pathology are highly desirable. In our study, fecal calprotectin concentrations significantly decreased during treatment in cats with CE. These findings are similar to those reported in a recent study of 17 cats with CE, 12 with FRE, 4 with IRE, and 1 with unspecified disease, all diagnosed retrospectively based on treatment response [20]. In this study, fecal calprotectin concentrations were decreased in cats that responded to treatment [20]. Our results are similar to those described in dogs with IBD, where fecal calprotectin concentrations significantly decreased after treatment, and dogs showed clinical improvement [25]. In another study in dogs with CE, fecal calprotectin concentrations were correlated with disease clinical activity [30]. The same findings also have been described in human patients, where fecal calprotectin has been used as a non‐invasive marker to monitor clinical disease severity and to differentiate active and quiescent Crohn's disease in adults and children [31, 32]. Based on the above, fecal calprotectin seems to be a promising biomarker for monitoring treatment in cats with CE. Further investigation is needed to determine if calprotectin concentration can be used for long‐term follow‐up of treatment.

In contrast to fecal calprotectin concentrations, serum calprotectin concentrations did not differ between cats with CE and healthy control cats at baseline and did not decrease in response to treatment in cats with CE. Our results are in contrast with a study in dogs with CIE, in which serum calprotectin concentrations were increased compared with controls [33], but similar to a more recent study in dogs, in which dogs with CIE had increased serum calprotectin concentrations that were not different from controls and did not decrease in response to treatment [25].

Our results are in contrast with those in humans with IBD (ulcerative colitis and Crohn's disease). In humans, serum calprotectin has gained more attention as a serum‐based biomarker for IBD, because in humans it may be more convenient in routine practice to acquire blood samples than fecal samples. In one study in humans with IBD, serum calprotectin concentration was significantly increased compared with controls [34]. In another study in humans, serum calprotectin concentration strongly correlated with fecal calprotectin and was the strongest predictor of IBD diagnosis in comparison with other biomarkers such as C‐reactive protein and albumin in 156 patients [35]. The same study concluded that a diagnostic and prognostic model with a combination of serum calprotectin and other blood‐based biomarkers is capable of predicting the inflammatory burden in IBD patients, as well as predicting disease and its outcome [35]. However, in a subset of 50 patients with paired serum and fecal calprotectin concentrations, fecal calprotectin was a better discriminating marker for the differentiation of IBD from controls [36]. The reasons for this apparent difference in the utility of serum calprotectin between species are not known.

Calprotectin is an inflammatory marker that is mainly produced by myeloid cells, predominately neutrophils, monocytes, and macrophages. It is a calcium‐ and zinc‐binding protein consisting of two small anionic proteins (S100A8 and S100A9), the S100A8/A9 protein complex, and it belongs to the damage‐associated molecular pattern (DAMP) molecules of the innate immune response. In IBD in humans, where the main histopathological finding is mucosal neutrophilic inflammation, fecal calprotectin has proven to be a promising biomarker. Studies in humans have correlated calprotectin with the severity of histologic lesions [37]. In cats with CE, the main type of inflammation is lymphocytic. Regardless, fecal calprotectin concentration was increased compared with controls in our study and in a previous study [19]. Our results support previous evidence suggesting that lymphocytes may express or be associated with calprotectin expression under certain pathological or inflammatory conditions, thus making calprotectin, and especially fecal calprotectin, a promising biomarker in these cases [38, 39, 40].

Although it is found in various body fluids, calprotectin concentration in feces is six times higher than in blood in healthy humans [27, 41], which may be one of the reasons why serum calprotectin concentration does not appear to be a good biomarker for cats with CE, compared with fecal calprotectin. In humans, the increase of calprotectin concentration in fecal samples during inflammatory diseases is proportional to its increase in other biological fluids in the body, but in our study, the results were in contrast with findings in humans. In a study in dogs, serum calprotectin concentration was increased with corticosteroid administration [31]. In this study, calprotectin concentrations were increased in serum after treatment with prednisolone or a combination of prednisolone and metronidazole, despite clinical improvement [33]. No studies are available regarding the effect of corticosteroids on serum calprotectin concentrations in cats, but considering the results in dogs, serum calprotectin concentrations should be interpreted with caution in animals receiving corticosteroids. In our study, 21 of 27 cats with CE (77%) received prednisolone for treatment of CIE or SCGL, which could have affected our results. However, the fact that no difference was found in serum calprotectin concentration in cats with CE compared to healthy control cats at baseline makes this possibility less likely. In addition, because treatment with prednisolone is standard care in cats with CIE or SCGL, a practical marker for treatment monitoring should not be affected by corticosteroid treatment.

Our study failed to find any differences in serum and fecal calprotectin concentrations between cats with CIE and cats with SCGL. This finding might be because CIE and SCGL represent different stages of the same condition and that SCGL is a disease process of CIE, instead of being distinct diseases [1]. Another possible explanation could be that in both conditions (CIE and SCGL) the predominant inflammatory cells that infiltrate the mucosa are lymphocytes, and thus the secretion of calprotectin could be equally influenced in both conditions. In our study, only one cat had eosinophilic CIE, whereas all of the other cats with CIE had lymphoplasmacytic enteritis. Our results are similar to those of a recent study in cats with CE, in which no difference in fecal calprotectin concentrations was found between cats with CIE and cats with SCGL [19].

Our study had some limitations. One limitation is the relatively small number of cats with CIE and SCGL and the even smaller number of cats that completed the 3‐month follow‐up period. This factor potentially could have led to type 2 statistical error. However, ours was a prospective study, with relatively strict inclusion criteria and a 3‐month follow‐up period, and therefore it was challenging to enroll larger numbers of animals. Another limitation of our study is that the lower detection limit of the assay used was relatively high. Regardless, fecal calprotectin concentrations were significantly higher in cats with CE compared with controls and lower in cats with CE after treatment compared with baseline.

Another potential limitation of our study was that cats enrolled in the control group were not age‐matched with the cats in the CE group. This factor might have affected our results, but it is currently unknown whether age affects calprotectin concentrations in serum or feces in cats. A study in healthy humans reported that fecal calprotectin concentrations increase with age in adults [42]. The age difference between CE cats and healthy cats could have influenced our results regarding fecal calprotectin concentrations. However, even in humans, no age‐specific reference intervals are available, and fecal calprotectin is considered a very useful noninvasive biomarker in adult humans at any age [32, 42]. Moreover, in our study, in cats with CE, fecal calprotectin concentrations decreased during treatment, suggesting that calprotectin concentrations were influenced by disease activity and not simply by the age of the cats.

Finally, several of the cats in our study did not consume the hydrolyzed protein diet (Anallergenic, Royal Canin). This problem is commonly encountered in clinical practice when attempting to change the diet of cats that are hyporexic. Although making diet transitions very gradually and trying different diets may help with diet acceptance, many of the cats enrolled in our study were very ill, and taking more time for dietary trials could have been detrimental to those cats.

In conclusion, fecal calprotectin concentration could be useful as a biomarker for the diagnosis and treatment monitoring in cats with CE because it was increased in cats with CE compared with healthy controls and decreased during treatment. Serum calprotectin concentrations failed to differentiate cats with CE from healthy control cats and were found to not be helpful for treatment monitoring in cats with CE. Neither fecal nor serum calprotectin concentrations could differentiate cats with CIE from cats with SCGL. Additional studies are needed to investigate the potential use of fecal calprotectin concentrations in cats with CE as a marker for response to treatment.

## Disclosure

Authors declare no off‐label use of antimicrobials.

## Ethics Statement

Study protocol approved by the Animal Ethics Committee of the University of Thessaly, Greece (AUP number:115/6.10.2020). Authors declare human ethics approval was not needed.

## Conflicts of Interest

Jonathan A. Lidbury, Jan S. Suchodolski and Joerg M. Steiner are employes at Gastroenterology (GI) Laboratory Texas A&M University that offers laboratory testing including calprotectin on a fee for service basis. Shelley Newman is a consultant as an anatomic pathologist at GI Laboratory Texas A&M University that offers histopathology and immunohistochemistry examination on a fee for service basis. All authors except Dimitra A. Karra were blinded on each cat's history and clinical signs, and each cats group.
